# Supplementary material for: Immunoreactive Trypsinogen and Free Carnitine Changes on Newborn Screening after Birth in Patients Who Develop Type 1 Diabetes
Source: Nutrients. 2021 Oct 19;13(10):3669. doi: 10.3390/nu13103669 (PMC8538382; doi:10.3390/nu13103669)
Supplement: Supplementary file 1 [file nutrients-13-03669-s001.zip › nutrients-1389313-supplementary.pdf]

Supplementary Table S1. Secondary analysis of other newborn screening analytes

**NBS analytes (unit/whole blood)**	Median and Interquartile Range	Median and Interquartile Range
**IRT (μg/L)**	**20.24 (16.15-29-52)**	**18.71 (13.96-26.92)**
**C10 (μmol/L)**	0.12 (0.09-0.16)	0.12 (0.08-0.16)
**C10:1(μmol/L)**	0.09 (0.06-0.13)	0.09 (0.06-0.12)
**C12(μmol/L)**	0.29 (0.16-0.47)	0.27 (0.17-0.49)
**C12:1(μmol/L)**	0.16 (0.12-0.45)	0.22 (0.09-0.41)
**C14(μmol/L)**	0.27 (0.22-0.36)	0.28 (0.21-0.39)
**C14:1(μmol/L)**	0.16 (0.12-0.24)	0.16 (0.11-0.25)
**C16(μmol/L)**	3.51 (2.63-4.52)	3.51 (2.79-4.79)
**C2(μmol/L)**	40.62 (30.84-52.10)	41.60 (31.77-54.09)
**C3(μmol/L)**	2.05 (1.56-2.79)	2.13 (1.51-3.02)
**C4(μmol/L)**	0.26 (0.19-0.34)	0.27 (0.20-0.37)
**C5(μmol/L)**	0.17 (0.12-0.26)	0.16 (0.11-0.26)
**C5D(μmol/L)**	0.04 (0.03-0.07)	0.05 (0.03-0.07)
**C5OH(μmol/L)**	0.16 (0.13-0.22)	0.17 (0.13-0.22)
**C6(μmol/L)**	0.06 (0.05-0.09)	0.06 (0.05-0.09)
**C8(μmol/L)**	0.08 (0.06-0.11)	0.08 (0.06-0.11)
**CAR(μmol/L)**	**25.50 (18.98-33.61)**	**27.26 (21.22-34.86)**
**CIT(μmol/L)**	16.61 (12.13-21.42)	16.09 (11.19-21.81)
**GLY(μmol/L)**	300.34 (242.04-379.79)	284.55 (230.27-370.10)
**LEU(μmol/L)**	222.93 (177.80-267.14)	226.10 (179.49-273.64)
**MET(μmol/L)**	27.07 (22.21-32.47)	26.97 (21.48-33.43)
**PHE(μmol/L)**	54.74 (48.31-65.14)	55.59 (47.80-66.39)
**TYR(μmol/L)**	82.24 (66.13-107.29)	82.00 (64.07-105.96)
**TSH mIU/L**	1.30 (0.64-2.33)	1.34 (0.69-2.35)

IRT-immunoreactive trypsinogen C10-decanoylcarnitine, C10:1-decenoylcarnitine, C12-dodecanoylcarnitine, C12:1-dodecenoylcarnitine, C14-tetradecanoylcarnitine, C14:1-tetradecenoylcarnitine, C16-hexadecanoylcarnitne, C2-acetylcarnitine, C3-proprionylcarnitine, C4-butyryl/isobutyrylcarnitine, C5-iso/valerylcarnitine, C5D-glutarylcarnitine, C5OH-methylmalonylcarnitine/hydroxyvalerylcarnitine, C6-hexanoylcarnitine, C8-octanoylcarnitine, CAR-free carnitine, CIT-citrulline, LEU-leucine/isoleucine, MET-methionine, PHE-phenylalanine, TYR-tyrosine, TSH-thyroid stimulating hormone

*Analyte values in bold: Statistically significant (CAR p value= 0.018, IRT unadjusted p value=0.045, adjusted p value=0.835)
